# Typical Carcinoid Tumor of the Lung: A Case Report Demonstrating Surveillance, Epidemiology, and End Results Database Analysis

**DOI:** 10.7759/cureus.117647

**Published:** 2026-10-08

**Authors:** Naveen Kulkarni, Ronda Albrecht

**Affiliations:** 1 Thoracic Surgery, Henry Ford Jackson Hospital, Jackson, USA

**Keywords:** atypical carcinoid, general thoracic surgery, neuroendocrine tumor, pulmonary carcinoid tumor, typical carcinoid

## Abstract

Typical lung carcinoid tumors are classified as well-differentiated, low-grade (G1) neuroendocrine neoplasms. This case study details the clinical course, diagnostic complexity, and definitive management of a 41-year-old African American male diagnosed with a histologically confirmed typical carcinoid (G1) that manifested a highly aggressive anatomical phenotype. Key features included bulky multi-station ipsilateral mediastinal lymph node metastasis (N2 disease), prevalent lymphovascular invasion, and direct phrenic nerve involvement. Following definitive surgical resection (left upper lobe segmentectomy), the disease was pathologically staged as pT2a N2 M0 (Stage IIIA).

## Introduction

Bronchopulmonary neuroendocrine neoplasms (BP-NENs) represent a rare yet highly heterogeneous category of lung malignancies, accounting for approximately 1% to 2% of all primary pulmonary tumors [1]. These tumors originate from the diffuse neuroendocrine cell system within the bronchopulmonary tract [2]. The current World Health Organization (WHO) classification system stratifies BP-NENs based on critical morphological indices, specifically the mitotic count and the presence or absence of tumor necrosis [3]. Pulmonary neuroendocrine neoplasms have four primary types: typical carcinoid (G1), atypical carcinoid (G2), large-cell neuroendocrine carcinoma (G3), and small-cell lung carcinoma (G3) [4].

Typical carcinoid tumors comprise 90% of all lung carcinoids [5]. Histologically, typical carcinoid is defined by a low mitotic rate (fewer than 2 mitoses per 2 mm²) and the complete absence of necrosis [3]. This biological characteristic underpins the exceptional long-term prognosis, with five-year survival rates ranging from 87% to 100% [3]. In contrast, atypical carcinoids are defined by heightened mitotic activity (2-10 mitoses per 2 mm²) and/or the presence of focal necrosis, resulting in a considerably poorer prognosis (five-year survival rate of 40% to 83%) [3].

This case is uncommon because it displayed histologic criteria for typical carcinoids, and yet behaved more aggressively. Tumor metastases occur in 5-20% of cases with typical carcinoids, compared with up to 70% of atypical carcinoids [3], demonstrating the rare behavior of certain typical carcinoids, including our case. This development is clinically significant as it changes prognosis and management for certain typical carcinoid subsets.

## Case presentation

This report presents a case that challenges the conventional expectation of low-grade tumor behavior. Low-grade tumors are more likely to have no regional metastatic lymphadenopathy [4]. The patient presented with a histologically low-grade typical carcinoid (G1) that exhibited a metastatic behavior characteristic of intermediate- or high-grade disease.

A 41-year-old African American male initially sought medical attention for non-specific right upper quadrant abdominal discomfort, which had been persistent for six months. Initial diagnostic imaging consisted of an ultrasound of the gallbladder followed by magnetic resonance imaging (MRI) of the abdomen and pelvis. A lung nodule was noted on the MRI of the abdomen/pelvis. A dedicated computed tomography (CT) scan of the chest was ordered, which identified a highly suspicious lesion measuring 2.4 × 2.7 cm; it was lobulated and pleura-based, located in the left lung apex. The scan also delineated a 1.4 cm enlarged node in the left aortopulmonary (AP) window, along with subcentimeter bilateral mediastinal nodes, which immediately raised significant suspicion for primary malignancy with regional metastatic spread.

The patient was referred for a positron emission tomography (PET) scan, suspecting Stage IIIA carcinoma. The PET scan demonstrated high metabolic activity (hypermetabolism) within the left upper lobe nodule (Figure 1) and within the aortopulmonary window lymph nodes (Figure 2). The absence of additional hypermetabolic lesions throughout the body confirmed the disease to be localized to the chest.

**Figure 1:**
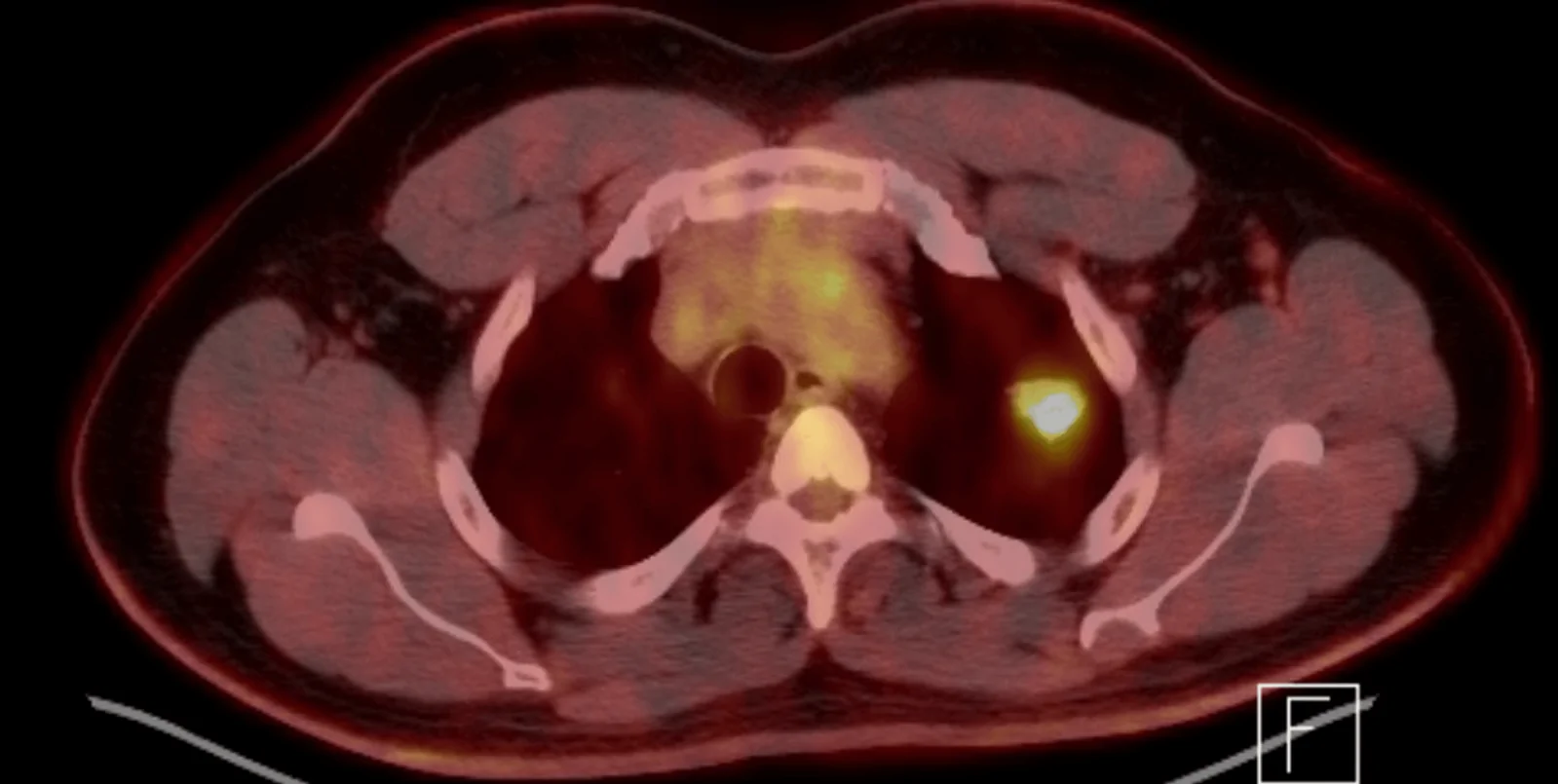
Computed tomography/positron emission tomography fusion axial image showing left upper nodule with high fluorodeoxyglucose-18 activity consistent with a neoplasm.

**Figure 2:**
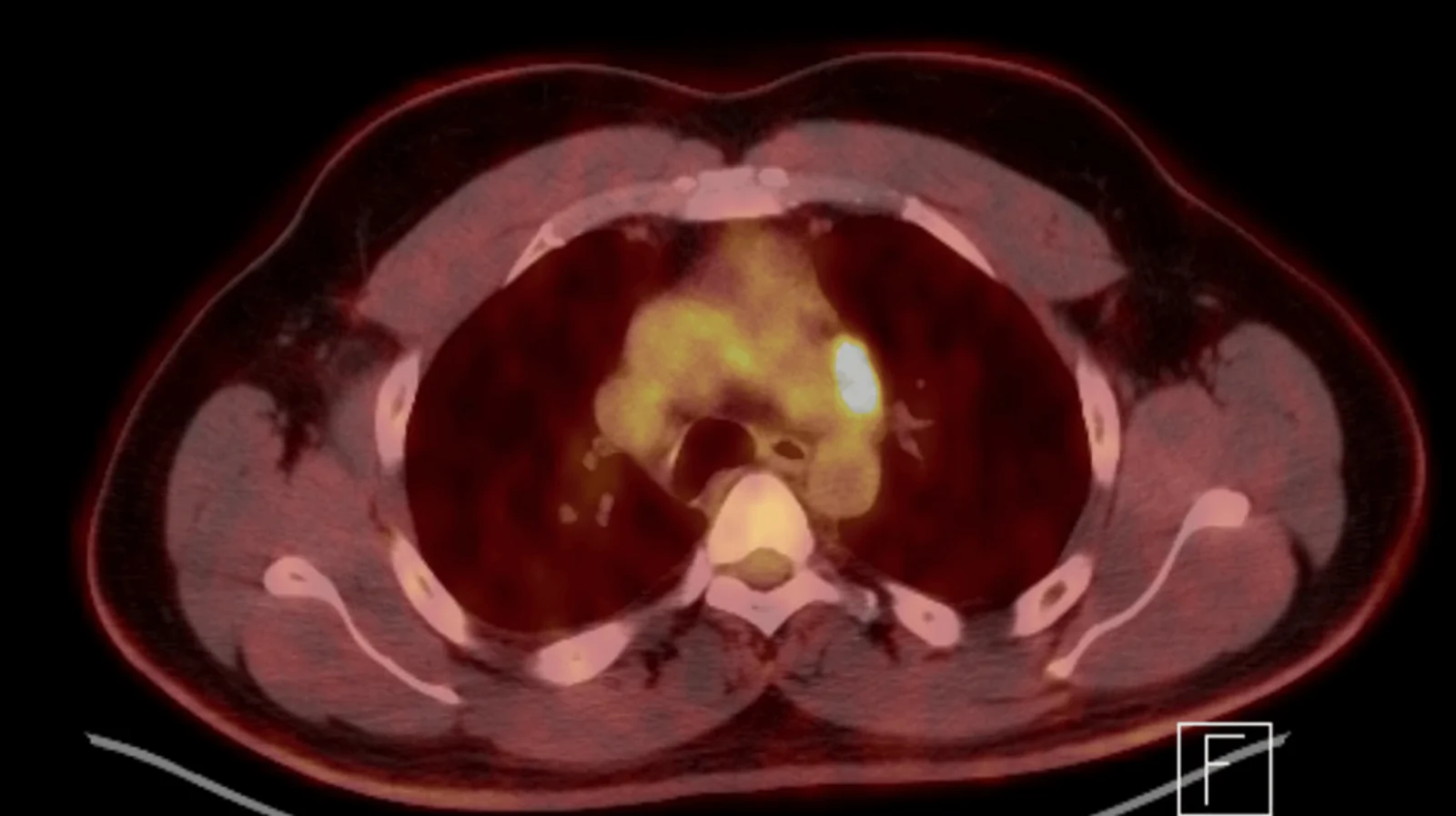
Computed tomography/positron emission tomography fusion axial image showing lymph nodes in the aortopulmonary window with high fluorodeoxyglucose-18 activity consistent with nodal metastasis.

Analysis of preoperative diagnostic procedures

Given the high radiological likelihood of malignancy and confirmed nodal hypermetabolism, the patient underwent a planned sequence of minimally invasive procedures including fiberoptic and navigational bronchoscopy, bronchoalveolar lavage, endobronchial ultrasound-guided transbronchial needle aspiration (EBUS-TBNA) of the left hilar lymph node, and transthoracic needle biopsy (TTNB) of the apical mass.

Cytologic evaluation of the bronchial lavage fluid was negative. EBUS-TBNA was done five times in nodal stations 4L, 7, and 10L. Cytologic TTNB provided non-diagnostic tissue, and the transthoracic biopsy provided no diagnostic pathologic tissue. Rapid on-site evaluation was not available at the institution. However, it would have enhanced our diagnostic yield [6]. Carcinoids frequently exhibit a dense stromal and desmoplastic composition, which results in low cellularity within small core needle biopsies and fine-needle aspiration samples. Given the PET findings, the index of suspicion for cancer was still high, and a more invasive diagnostic procedure was planned (left video thoracoscopy).

Definitive surgical and pathological findings

The patient proceeded with a robot-assisted video-assisted thoracoscopic surgery (VATS), which included biopsy of multiple mediastinal lymph node stations. Intraoperatively, the surgeon noted that several lymph node stations were markedly enlarged and “bulky,” leading to a strong suspicion of extensive metastatic disease. Frozen section analysis confirmed neoplastic involvement within the nodes, prompting the precautionary placement of a venous access port in anticipation of required systemic chemotherapy, which reflected the great intraoperative concern for advanced, aggressive disease.

Final pathological evaluation provided the diagnosis and staging. The primary resected tumor was confirmed as a 3.8 cm typical carcinoid tumor with negative surgical margins. The analysis revealed multiple adverse prognostic features, including confirmed lymphovascular invasion (LVI) and significant metastatic involvement in multiple ipsilateral mediastinal lymph node stations (stations 5 and 6).

Final pathology on the nodes confirmed typical carcinoid by the immunohistochemical stain (Figure 3) and the hematoxylin and eosin stain (Figure 4). Tumor board discussion prompted reoperation for local/regional control. The patient was an avid weightlifter and athlete and wanted maximum lung preservation, and PET and CT did not show hilar lymphadenopathy. For this reason, a segmentectomy was chosen over a lobectomy. The resection margin on the segmentectomy was 1 cm after staple removal. This is considered an R0 resection. Follow-up CT of the chest after six months showed no active disease. Robot-assisted left VATS with left upper lobe segmentectomy and additional nodal sampling was performed in lymph node stations 8, 9, and 10 (but were negative). The final pathological staging was pT2a N2 M0 (Stage IIIA). LVI and phrenic nerve involvement were confirmed.

**Figure 3:**
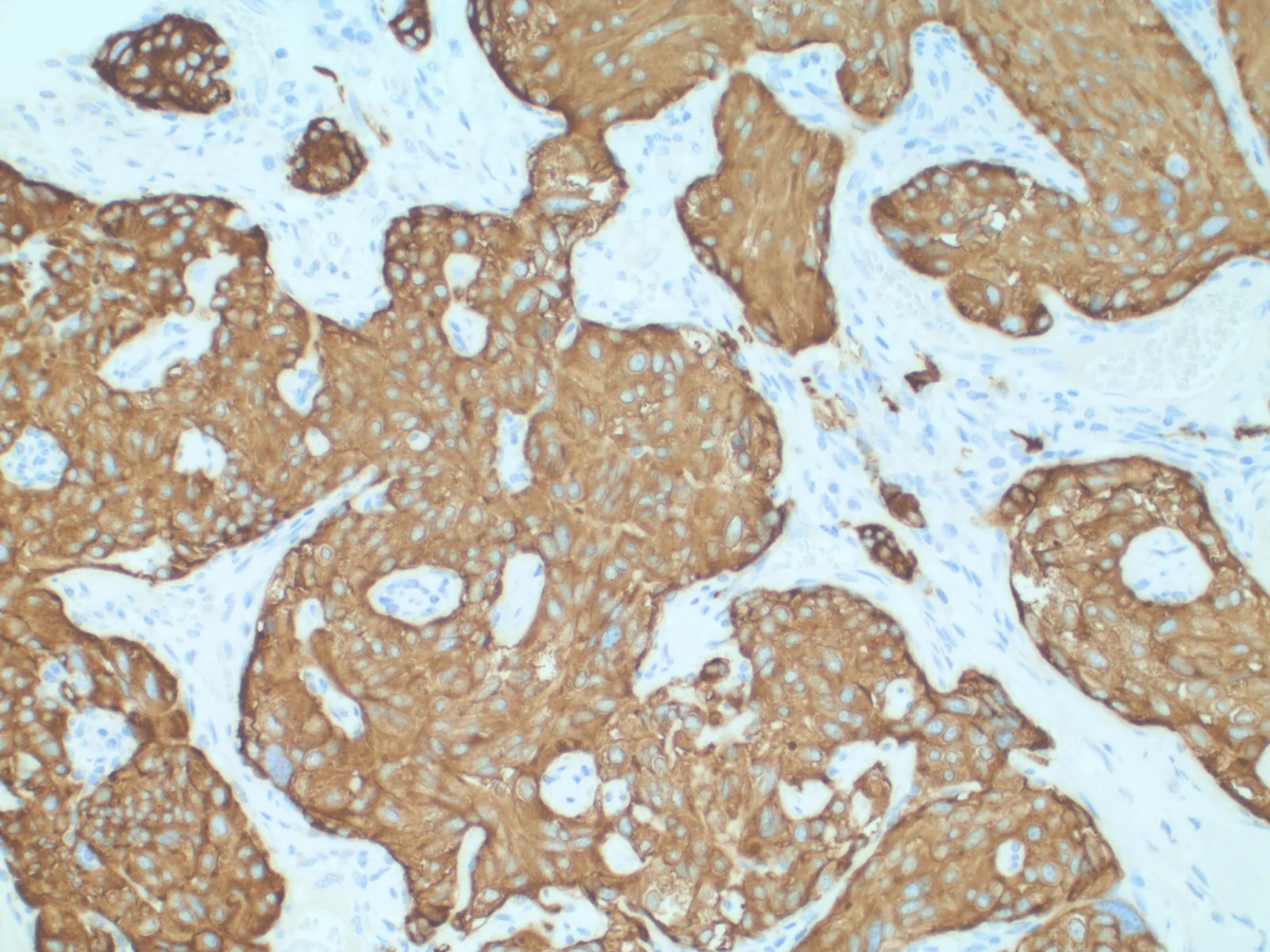
Synaptophysin stain (200×) diffusely lighting up the carcinoid tumor.

**Figure 4:**
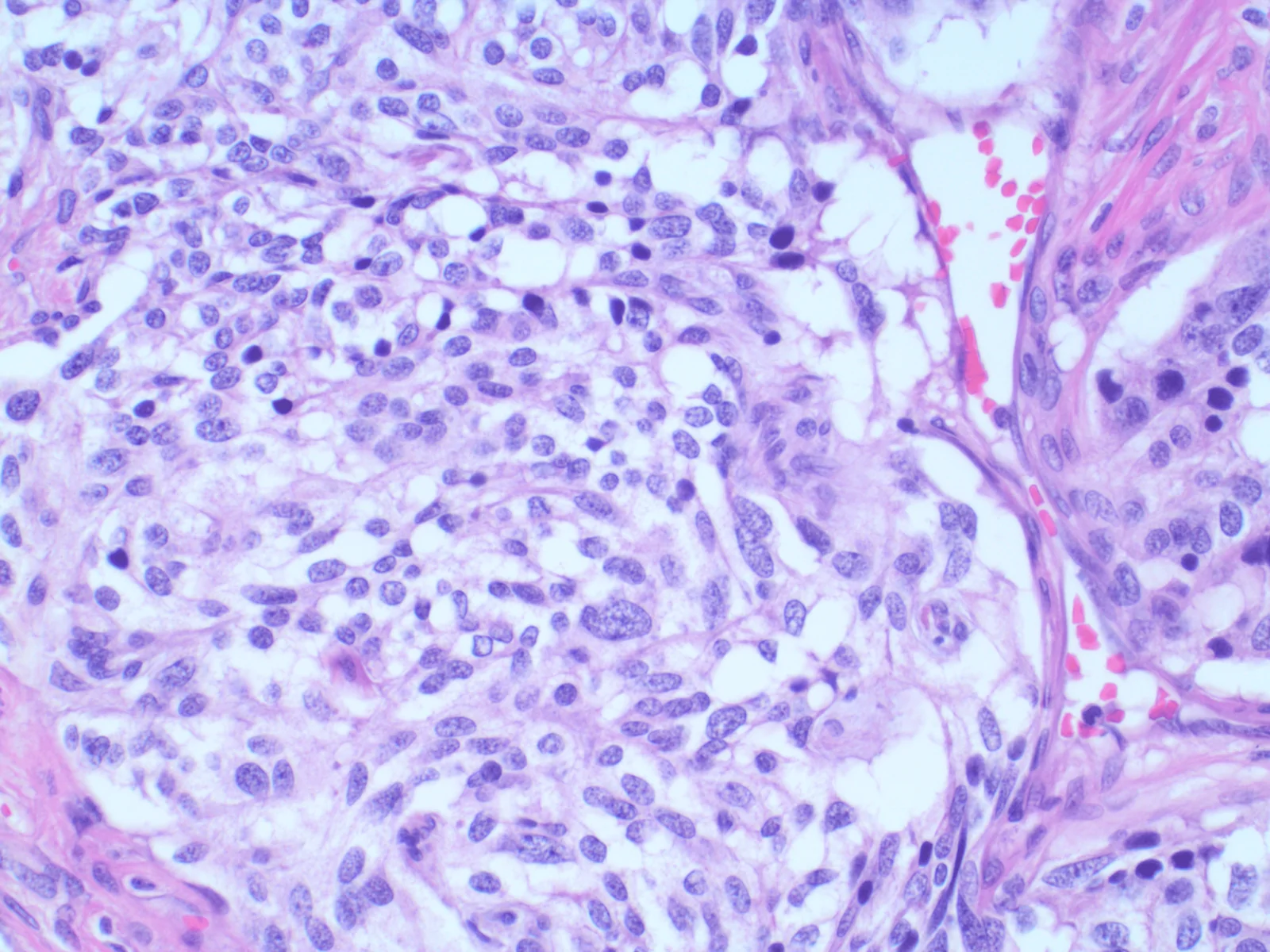
Hematoxylin and eosin stain (200×) demonstrating uniform cells arranged in cords with salt-and-pepper chromatin and moderate cytoplasm.

Pathologic staging: justification of pT2a N2 M0 (Stage IIIA)

Second tumor board discussion led to the following recommendations: A 1,4,7,10-tetraazacyclododecane-1,4,7,10-tetraacetic acid conjugated to tyrosine-3-octreotate (DOTATATE) scan, along with close follow-up without adjuvant chemotherapy or radiation, given the indolent nature of typical carcinoid despite N2 disease, as all gross disease was resected. Due to long waiting times for the test, DOTATATE was not performed six months after surgery.

## Discussion

The presence of nodal involvement (N+) is an important clinical prognostic feature [7]. Lymph node metastases diminish the five-year disease-free survival (DFS) rate to approximately 73.3% compared to 93.8% in node-negative patients [8]. The multi-station N2 disease is a prognostic indicator of worse survival rates and is much more prevalent in atypical carcinoids. The high-risk features observed, namely, multi-station N2 metastasis, LVI, and perineural invasion, are usually highly suggestive of intermediate-grade (AC, G2) biological behavior [9]. This tumor was pathologically confirmed as G1 (low mitoses, no necrosis), yet achieved such a degree of spread, suggesting that the standard WHO morphological criteria alone may be insufficient to fully capture the inherent metastatic competence of certain typical carcinoid subsets in which chromosomal instability, epigenetic regulators, and variations in chromatin remodeling have been associated with more aggressive behavior [10].

For this patient, the multidisciplinary tumor board concluded that adjuvant chemotherapy was not indicated. This approach aligns precisely with current international consensus guidelines, including the National Comprehensive Cancer Network (NCCN), which recommends against postoperative adjuvant cytotoxic therapy for resected bronchial neuroendocrine tumors, even in the explicit setting of positive lymph nodes (pN+) [11]. Locoregional radiation therapy was also debated, but was not given at this time. If recurrence is noted on subsequent CT scans, then radiation may be considered.

Follow-up

According to the multidisciplinary tumor board and NCCN guidelines for pT2a N2 M0 TC, close follow-up with CT scans at six-month intervals for the first two years, and a DOTATATE scan when available, was recommended rather than adjuvant therapy [12]. At the first six-month interval, the patient did not show local or distant metastases, as seen on a contrast-enhanced CT scan of the chest.

The classification of the disease as Stage IIIA, compounded by high-risk features (N2, LVI), necessitates lifelong oncological surveillance. Late recurrences are a well-documented phenomenon in BP-NENs, making follow-up essential [13].

The cornerstone surveillance tool recommended in this case is the gallium-68 (Ga) DOTATATE PET scan. This advanced functional imaging modality is specifically recommended for both preoperative staging and for optimized postoperative follow-up. By employing Ga-DOTATATE PET/CT, the surveillance strategy is maximally equipped to detect early local recurrence in the high-risk surgical field or the emergence of occult distant metastases, thereby ensuring timely therapeutic intervention should the aggressive phenotype resurface [14].

## Conclusions

This case report details a histologically verified typical lung carcinoid (G1) that presented with a clinically aggressive Stage IIIA profile (pT2a N2 M0), characterized by extensive multi-station nodal metastasis, LVI, and phrenic nerve involvement. This presentation highlights a rare subset of typical carcinoid that deviates significantly from the conventional definition of indolence and underscores the functional limitations of relying exclusively on morphological grade criteria for fully assessing metastatic potential. The final therapeutic plan, which centered on complete anatomical surgical resection followed by the calculated omission of adjuvant systemic cytotoxic chemotherapy, reflects a rigorous adherence to the evidence-based consensus within current thoracic oncology guidelines. The decision to defer chemotherapy is scientifically sound, recognizing that the favorable G1 tumor biology confers inherent resistance to cytotoxic agents such as capecitabine and temozolomide and platinum-based regimens, irrespective of the high anatomical burden imposed by Stage IIIA disease. The long-term management strategy for this high-risk typical carcinoid relies instead upon maximizing locoregional control (aggressive surgery, potential targeted radiotherapy) and instituting rigorous, high-sensitivity, long-term surveillance using Ga-DOTATATE PET/CT to monitor for disease recurrence.
